# Supplementary material for: Element Levels and Predictors of Exposure in the Hair of Ethiopian Children
Source: Int J Environ Res Public Health. 2020 Nov 21;17(22):8652. doi: 10.3390/ijerph17228652 (PMC7700284; doi:10.3390/ijerph17228652)
Supplement: Supplementary file 1 [file ijerph-17-08652-s001.pdf]

International Journal of
Environmental Research and Public Health

MDPI

Supplementary Materials: Element Levels and Predictors of Exposure in the Hair of Ethiopian Children

Maria Luisa Astolfi [1,*], Georgios Pietris [2], Corrado Mazzei [3], Elisabetta Marconi [4] and Silvia Canepari [1]

1. Department of Chemistry, Sapienza University, Piazzale Aldo Moro 5, I-00185 Rome, Italy; silvia.canepari@uniroma1.it
2. Department of General Surgery, Thoracic Diseases General Hospital Sotiria of Athens, Mesogion 152, 115 27 Athens, Greece; gpietris@gmail.com
3. Canon Toshiba Medical Systems s.r.l., Via Canton 115, I-00144 Rome, Italy; corradomazzei1964@gmail.com
4. Department of Public Health and Infectious Diseases, Sapienza University, Piazzale Aldo Moro 5, I-00185 Rome, Italy; elisabetta.marconi@uniroma1.it

* Correspondence: marialuisa.astolfi@uniroma1.it@uniroma1.it ; Tel.: +39-064-991-3384

Section 1. Reagents, standards, and calibration

Analytical reagent grade concentrated HNO_3 (67–70%; super-pure) was obtained from Carlo Erba Reagents S.r.l. (Milan, Italy) and HCl (assay >36%; residue <3 mg L^{-1}) and H_2O_2 (assay >30%) were obtained from Promochem, LGC Standards GmbH (Wesel, Germany). The 5% HCl was used as a carrier and 0.05% $NaBH_4$ (Sigma-Aldrich Chemie GmbH, St. Louis, USA) in 0.05% NaOH (assay >98%, anhydrous pellets, RPE for analysis, ACS – ISO; Carlo Erba Reagents, Milan, Italy) as reducing agent for cold vapor atomic fluorescence spectrometry (CV-AFS). Deionized water with a resistivity ≤18.3 MΩ cm was obtained using an Arioso Power I RO-UP Scholar UV water purification system (Human Corporation, Songpa-Ku, Seoul, Korea).

All calibration standard solutions for inductively coupled mass spectrometry (ICP-MS) were prepared from a multi-element standard solution (1.000 ± 0.005 mg L^{-1} As, Al, Ba, Be, Bi, Cd, Cr, Cs, Cu, Ga, La, Li, Mn, Mo, Nb, Ni, Pb, Rb, Sb, Se, Sn, Te, Ti, Tl, U, V, W, and Zr; 5.00 ± 0.03 mg L^{-1} Ce and Co; 10.00 ± 0.05 mg L^{-1} Fe and Zn; 50.00 ± 0.25 mg L^{-1} P and Si; 55.00 ± 0.25 mg L^{-1} B and Sr; 500.0 ± 2.5 mg L^{-1} K, Mg, and Na; 1000 ± 5 mg L^{-1} Ca and S; Ultra Scientific/Agilent Technologies, North Kingstown, RI, USA) and for CV-AFS from the Hg standard solution (1002 ± 7 mg L^{-1}; SCP Science, Baie D'Urfé, Canada) by dilution with 3% (v/v) HNO_3 (same percentage of acid present in the sample) in deionized water.

^{89}Y (0.005 mg L^{-1}) and ^{45}Sc, ^{103}Rh, ^{115}In, and ^{232}Th (0.01 mg L^{-1}) were chosen as the internal standard for all the ICP-MS measurements to control the nebulizer efficiency. A multi-element internal standard solution containing 0.005 mg L^{-1} of Y and 0.010 mg L^{-1} of Sc, Rh, In, and Th was prepared from single element stock standards (1000 ± 2 mg L^{-1}; Panreac Química, Barcelona, Spain, and 1000 ± 5 mg L^{-1}; Merck KGaA, Darmstadt, Germany, respectively) in HNO_3 1% (v/v). A standard solution containing 0.005 mg L^{-1} Ba, Be, Ce, Co, In, Pb, Mg, Tl, and Th was prepared daily in HNO_3 1% (v/v) from a multi-standard stock solution (10.00 ± 0.05 mg L^{-1}; Spectro Pure, Ricca Chemical Company, Arlington, TX, USA) to select the best ICP-MS performance.

The calibration curves for the analytes by ICP-MS were prepared using seven different concentrations in the range of 0.0005–0.05 mg L^{-1} for Al, As, Ba, Be, Bi, Cd, Ce, Cr, Cs, Cu, Ga, La, Li, Mn, Mo, Nb, Ni, Pb, Rb, Sb, Se, Sn, Te, Ti, Tl, U, V, W, and Zr; 0.0025–0.25 mg L^{-1} for Co, Fe, and Zn; 0.0275–2.75 mg L^{-1} for B, P, Si, and Sr; 0.25–25 mg L^{-1} for K, Mg and Na; and 0.5–50 mg L^{-1} for Ca. Hg was determined by CV-AFS using nine different standard concentrations ranging from 0.01 to 1.5 μg L^{-1}. All measurements were performed using a full quantitative mode analysis. The correlation coefficients for all calibration curves were at least 0.999, showing good linear relationships throughout the ranges of the concentrations studied. Moreover, the linear concentration range was verified using at least five levels (including zero) by Mandel fitting test.

International Journal of Environmental Research and Public Health

Table S1. Isotopes, analysis mode, and internal standards.

Isotope/ Element[a]	ICP-MS Mode	Internal standard
**^{27}Al**	Standard	^{45}Sc
**^{75}As**	CRI[b]	^{79}Y
**^{11}B**	Standard	^{45}Sc
**^{137}Ba**	Standard	^{115}In
**^{9}Be**	Standard	^{45}Sc
**^{209}Bi**	Standard	^{232}Th
**^{44}Ca**	Standard	^{79}Y
**^{112}Cd**	Standard	^{115}In
**^{140}Ce**	Standard	^{115}In
**^{59}Co**	Standard	^{45}Sc
**^{52}Cr**	CRI[b]	^{79}Y
**^{133}Cs**	Standard	^{115}In
**^{65}Cu**	Standard	^{79}Y
**^{56}Fe**	CRI[b]	^{79}Y
**^{71}Ga**	Standard	^{79}Y
**Hg**	-	-
**^{39}K**	Standard	^{45}Sc
**^{139}La**	Standard	^{115}In
**^{7}Li**	Standard	^{45}Sc
**^{24}Mg**	Standard	^{45}Sc
**^{55}Mn**	CRI[b]	^{79}Y
**^{98}Mo**	Standard	^{103}Rh
**^{23}Na**	Standard	^{45}Sc
**^{93}Nb**	Standard	^{103}Rh
**^{60}Ni**	Standard	^{45}Sc
**^{31}P**	Standard	^{45}Sc
**^{208}Pb**	Standard	^{232}Th
**^{85}Rb**	Standard	^{79}Y
**^{121}Sb**	Standard	^{115}In
**^{76}Se**	CRI[b]	^{79}Y
**^{28}Si**	Standard	^{45}Sc
**^{118}Sn**	Standard	^{115}In
**^{88}Sr**	Standard	^{79}Y
**^{125}Te**	Standard	^{115}In
**^{49}Ti**	Standard	^{45}Sc
**^{205}Tl**	Standard	^{232}Th
**^{238}U**	Standard	^{232}Th
**^{51}V**	CRI[b]	^{79}Y
**^{182}W**	Standard	^{232}Th
**^{66}Zn**	Standard	^{79}Y
**^{90}Zr**	Standard	^{79}Y

[a] A cold vapor atomic fluorescence spectrometry was used for Hg determination, while inductively coupled mass spectrometry was used for the other elements. [b] Collision-reaction interface.

International Journal of
Environmental Research and Public Health

MDPI

Table S2. Element concentrations (mg kg^{-1}) in children's hair by age group.

	<5 years			6-11 years			12-18 years		
**Element**	**GM**	**Min**	**Max**	**GM**	**Min**	**Max**	**GM**	**Min**	**Max**
**Al**	7	<DL	78	1	<DL	233	1	<DL	58
**As**	0.03	<DL	0.25	0.05	<DL	0.65	0.03	<DL	0.17
**B**	1.2	<DL	5.1	<DL	<DL	6.2	<DL	<DL	29.2
**Ba**	9	0	73	12	0.2	74	13	2	45
**Be**	**0.004**[a]	<DL	0.173	**0.013**[b]	<DL	0.181	0.011	<DL	0.049
**Bi**	0.001	<DL	0.025	0.001	<DL	0.551	<DL	<DL	0.076
**Ca**	1990	310	17300	1230	<DL	10700	1580	451	3590
**Cd**	0.09	0.03	0.37	0.10	<DL	1.58	0.12	0.01	0.56
**Ce**	**0.6**[a]	0.03	13.4	**1.6**[b]	0.2	12.9	1.1	0.2	2.8
**Co**	**0.23**[a]	0.02	3.23	**0.44**[b]	0.02	3.58	0.30	0.09	0.78
**Cr**	0.5	<DL	6.1	0.7	0.1	5.2	0.5	0.1	6.5
**Cs**	0.008	<DL	0.156	0.017	<DL	0.150	0.015	0.002	0.056
**Cu**	10.3	6.5	17.7	12.1	4.9	32.7	11.8	8.8	17.0
**Fe**	**151**[a]	11	3730	**365**[b]	27	4000	239	40	717
**Ga**	0.05	0.005	1.00	0.10	0.01	1.07	0.07	0.01	0.19
**Hg**	0.056	0.012	0.483	0.066	0.017	0.367	0.047	0.013	0.249
**K**	636	52	5790	1280	<DL	6690	1360	155	5880
**La**	**0.3**[a]	0.01	7.3	**0.8**[b]	0.1	6.6	0.6	0.1	1.4
**Li**	**0.13**[a]	0.02	1.34	**0.26**[b]	0.04	1.60	0.21	0.04	1.43
**Mg**	258	44	2320	306	62	1600	284	97	649
**Mn**	19	1	184	35	2	167	30	7	81
**Mo**	**0.03**[a]	<DL	1.53	**0.09**[b]	0.001	0.89	0.12	<DL	0.68
**Na**	**897**[a]	160	7450	**2510**[b]	361	15000	**2200**[b]	180	7120
**Nb**	0.00	<DL	0.80	0.001	<DL	0.88	0.001	<DL	0.08
**Ni**	1.0	0.2	6.9	1.5	0.3	6.6	1.1	0.2	8.8
**P**	97	21	611	94	2	407	104	28	194
**Pb**	3.3	0.5	32.8	3.5	0.6	15.5	2.7	0.3	26.6
**Rb**	0.9	0.1	7.3	1.6	0.2	8.6	1.5	0.3	4.7
**Sb**	0.11	<DL	8.85	0.06	<DL	0.49	0.06	<DL	0.62
**Se**	0.23	<DL	0.67	0.17	<DL	0.59	0.19	0.10	0.40
**Si**	18	<DL	472	110	3	499	123	3	484
**Sn**	0.27	0.04	1.05	0.20	<DL	1.01	0.25	0.05	2.38
**Sr**	17	3	99	19	3	76	21	4	44
**Te**	0.0015	<DL	0.0076	0.0014	<DL	0.0148	<DL	<DL	0.0077
**Ti**	11	2	226	20	2	253	13	3	43
**Tl**	0.0044	<0.0017	0.0243	0.0064	0.0020	0.0262	0.0054	0.0023	0.0104
**U**	0.020	0.002	0.167	0.037	0.002	0.204	0.030	0.010	0.099
**V**	0.6	0.1	10.3	1.1	<DL	12.0	0.7	0.2	2.6
**W**	<0.0017	<DL	0.0210	<DL	<DL	0.0462	<DL	<DL	<DL
**Zn**	112	39	300	67	<DL	451	98	10	234
**Zr**	0.29	0.02	6.05	0.53	0.01	6.29	0.35	0.06	1.54

[a, b] Nonparametric Kruskal-Wallis test was applied. Dunn's multiple comparison-adjusted by Bonferroni correction was used to make pairwise comparisons; different letters "a" and "b" in the same row indicate significant differences ($p < 0.05$ for Be, Ce, Co, Fe, La, Li, and Mo, and $p < 0.01$ for Na).

Table S3. Element levels (mg kg^{-1}) in male hair according to age.

	<5 years			6-11 years			12-18 years		
**Element**	**GM**	**Min**	**Max**	**GM**	**Min**	**Max**	**GM**	**Min**	**Max**
**Al**	16	<DL	78	1	<DL	233	1	<DL	41
**As**	0.03	<DL	0.21	0.05	<DL	0.65	0.04	<DL	0.17
**B**	1.1	<DL	5.1	1.1	<DL	2.4	1.0	<DL	1.0
**Ba**	6	<DL	20	8	<DL	74	12	2	39
**Be**	**0.001**[a]	<DL	0.054	**0.006**[b]	<DL	0.065	**0.008**[b]	<DL	0.033
**Bi**	0.001	<DL	0.013	<0.001	<DL	0.006	0.001	<DL	0.076
**Ca**	1460	345	7570	658	<DL	5710	1500	451	3600
**Cd**	0.08	0.03	0.22	0.04	<DL	0.34	0.08	0.01	0.26
**Ce**	**0.3**[a]	0.03	4.4	**1.0**[b]	0.2	6.2	0.9	0.2	2.3
**Co**	0.11	0.02	0.90	0.27	0.02	1.38	0.28	0.09	0.57
**Cr**	0.3	<DL	4.0	0.5	0.1	4.5	0.6	0.2	6.5
**Cs**	0.004	<DL	0.062	0.010	<DL	0.085	0.013	0.002	0.056
**Cu**	9.8	6.5	17.7	11.1	5.2	25.8	11.5	8.8	16.0
**Fe**	**66**[a]	11	1070	**221**[b]	27	1620	212	40	670
**Ga**	0.02	0.01	0.29	0.07	0.01	0.48	0.06	0.01	0.19
**Hg**	0.040	0.016	0.196	0.078	0.019	0.367	0.051	0.013	0.128
**K**	425	52	4420	683	<DL	3660	911	155	3170
**La**	**0.1**[a]	0.01	2.3	**0.5**[b]	0.1	3.3	0.5	0.1	1.2
**Li**	0.08	0.02	0.62	0.18	0.04	0.70	0.20	0.04	1.43
**Mg**	169	44	613	203	62	746	263	97	589
**Mn**	11	1	53	21	2	71	23	7	56
**Mo**	**0.02**[a]	0.01	0.22	0.05	<DL	0.22	**0.08**[b]	<DL	0.68
**Na**	794	160	7450	1740	361	10200	1450	180	3780
**Nb**	<DL	<DL	0.14	<0.0014	<DL	0.17	<0.0015	<DL	0.08
**Ni**	0.6	0.2	4.6	1.3	0.3	6.6	1.0	0.2	8.8
**P**	81	36	224	62	<DL	239	93	28	146
**Pb**	3.4	0.5	32.8	2.3	0.6	7.2	1.9	0.3	9.1
**Rb**	0.6	0.1	4.5	1.1	0.2	3.9	1.2	0.3	3.3
**Sb**	0.10	<DL	8.85	0.02	<DL	0.15	0.06	<DL	0.62
**Se**	0.21	<DL	0.52	0.16	<DL	0.59	0.22	0.16	0.40
**Si**	8	<DL	458	102	<DL	499	103	<DL	465
**Sn**	0.26	0.07	1.05	0.12	<DL	0.81	0.21	0.05	2.38
**Sr**	13	3	44	14	3	42	19	4	37
**Te**	<DL	<DL	0.0026	<DL	<DL	0.0033	<DL	<DL	0.0077
**Ti**	**5**[a]	2	58	**13**[b]	2	76	12	3	41
**Tl**	0.0037	0.0018	0.0126	0.0054	0.0028	0.0125	0.0048	0.0023	0.0092
**U**	**0.011**[a]	0.002	0.067	0.025	0.002	0.165	**0.030**[b]	0.010	0.099
**V**	0.3	0.1	2.8	0.6	<DL	4.1	0.7	0.2	1.7
**W**	<DL	<DL	0.0053	<0.0015	<DL	0.0128	<DL	<DL	<0.0014
**Zn**	137	81	300	72	<DL	451	95	10	202
**Zr**	0.12	0.03	0.87	0.34	0.01	1.90	0.32	0.06	1.30

[a, b] Nonparametric Kruskal-Wallis test was applied. Dunn's multiple comparison-adjusted by Bonferroni correction was used to make pairwise comparisons; different letters "a" and "b" in the same row indicate significant differences ($p < 0.05$).

Table S4. Element levels (mg kg^{-1}) in male hair according to age.

Element	<5 years			6-11 years			12-18 years		
	GM	Min	Max	GM	Min	Max	GM	Min	Max
Al	3	<DL	72	1	<DL	167	<DL	<DL	58
As	0.03	<DL	0.25	0.06	<DL	0.32	<DL	<DL	0.08
B	1.4	<DL	4.6	1.3	<DL	6.2	1.6	<DL	29.2
Ba	14	1	73	17	4	68	13	4	45
Be	0.018	0.001	0.173	0.024	0.005	0.181	0.014	0.004	0.049
Bi	0.002	<DL	0.025	0.003	<DL	0.551	0.002	<DL	0.018
Ca	2870	310	17300	2070	284	10700	1660	850	3030
Cd	0.10	0.04	0.37	0.20	0.03	1.58	0.18	0.05	0.56
Ce	1.6	0.2	13.4	2.3	0.6	12.9	1.3	0.5	2.8
Co	0.57	0.10	3.23	0.67	0.18	3.58	0.32	0.17	0.78
Cr	1.2	0.1	6.1	0.8	0.3	5.2	0.5	0.1	2.6
Cs	0.016	<0.0014	0.156	0.028	0.007	0.150	0.017	0.005	0.056
Cu	10.9	7.8	13.6	13.0	4.9	32.7	12.2	9.0	17.0
Fe	413	29	3730	553	130	4000	271	106	717
Ga	0.14	0.01	1.00	0.15	0.04	1.07	0.08	0.03	0.19
Hg	0.083	0.012	0.483	0.057	0.017	0.317	0.043	0.015	0.249
K	1030	178	5790	2150	382	6690	2030	604	5880
La	0.9	0.1	7.3	1.2	0.3	6.6	0.7	0.3	1.4
Li	0.23	0.02	1.34	0.36	0.12	1.60	0.23	0.09	0.47
Mg	429	114	2320	430	163	1600	307	162	649
Mn	38	3	184	54	18	167	38	13	81
Mo	0.05	<DL	1.53	0.15	0.01	0.89	0.17	0.07	0.53
Na	**1040**[a]	247	3420	**3410**[b]	619	150000	**3350**[b]	1490	7120
Nb	0.01	<DL	0.80	0.00	<DL	0.88	<DL	<DL	0.07
Ni	1.8	0.3	6.9	1.7	0.5	5.9	1.1	0.5	4.6
P	119	21	611	135	55	407	117	75	194
Pb	3.0	0.9	15.8	5.0	1.4	15.5	4.0	1.9	26.6
Rb	1.5	0.3	7.3	2.2	0.5	8.6	1.9	0.9	4.7
Sb	0.11	0.01	0.35	**0.13**[a]	0.03	0.49	**0.07**[b]	0.03	0.14
Se	0.25	0.13	0.67	0.18	<DL	0.58	0.17	0.10	0.28
Si	49	<DL	472	117	<DL	338	147	39	484
Sn	0.28	0.04	0.98	0.31	0.11	1.01	0.30	0.09	0.78
Sr	25	5	99	25	8	76	23	10	44
Te	0.0021	<DL	0.0076	0.0017	<DL	0.0148	0.0014	<DL	0.0064
Ti	26	2	226	27	6	253	13	5	43
Tl	0.0055	<0.0017	0.0243	0.0075	0.0020	0.0262	0.0061	0.0045	0.0104
U	0.042	0.006	0.167	0.053	0.013	0.204	0.031	0.011	0.090
V	1.5	0.2	10.3	1.8	0.4	12.0	0.8	0.4	2.6
W	0.0010	<DL	0.0210	<DL	<DL	0.0462	<DL	<DL	<DL
Zn	88	39	240	62	<DL	251	100	42	234
Zr	0.89	0.02	6.05	0.77	0.16	6.29	0.39	0.16	1.54

[a, b] Nonparametric Kruskal-Wallis test was applied. Dunn's multiple comparison-adjusted by Bonferroni correction was used to make pairwise comparisons; different letters "a" and "b" in the same row indicate significant differences (for Sb: $p < 0.05$, and for Na: $p < 0.05$ between 0-5 and 12-18 years old groups and $p < 0.01$ between <5 and 6-11 years old groups).

International Journal of Environmental Research and Public Health

MDPI

Table S5. Element levels (mg kg^{-1}) in children's hair according to passive smoking.

Element	Non-passive smoking (n = 63)			Passive smoking (n = 14)			p-value
	GM	Min	Max	GM	Min	Max	
**Al**	1	<DL	233	1	<DL	73	-
**As**	0.04	<DL	0.65	0.04	<DL	0.26	-
**B**	1.3	<DL	29.2	1.1	<DL	2.5	-
**Ba**	11	<DL	74	14	3	55	ns
**Be**	0.009	<DL	0.181	0.009	<DL	0.127	ns
**Bi**	0.001	<DL	0.551	0.001	<DL	0.039	-
**Ca**	1350	<DL	17300	2560	772	8870	ns
**Cd**	0.10	<DL	1.58	0.10	0.03	0.51	ns
**Ce**	1.1	0.03	13.4	1.0	0.1	9.4	ns
**Co**	0.32	0.02	3.58	0.35	0.05	3.09	ns
**Cr**	0.6	0.1	6.5	0.5	<DL	3.1	ns
**Cs**	0.013	<DL	0.156	0.016	0.001	0.098	ns
**Cu**	**12.0**	4.9	32.7	**9.4**	5.2	16.0	*
**Fe**	244	11	4000	289	16	2810	ns
**Ga**	0.07	0.010	1.07	0.08	0.01	0.71	ns
**Hg**	0.054	0.013	0.367	0.073	0.012	0.483	ns
**K**	1100	<DL	6690	936	118	4120	ns
**La**	0.6	0.01	7.3	0.6	0.0	4.7	ns
**Li**	0.20	0.02	1.60	0.21	0.02	1.43	ns
**Mg**	279	60	2320	318	44	1400	ns
**Mn**	27	1	184	32	4	135	ns
**Mo**	0.08	<DL	1.53	0.06	<DL	0.39	ns
**Na**	1940	180	15000	1300	160	5540	ns
**Nb**	0.001	<DL	0.88	0.002	<DL	0.66	-
**Ni**	1.2	0.2	8.8	1.2	0.2	4.6	ns
**P**	96	<DL	611	109	47	259	ns
**Pb**	3.3	0.3	32.8	2.6	0.5	11.3	ns
**Rb**	1.4	0.1	8.6	1.2	0.2	4.9	ns
**Sb**	0.07	<DL	8.85	0.06	<DL	0.35	ns
**Se**	0.19	<DL	0.59	0.18	0.02	0.67	ns
**Si**	67	<DL	499	73	<DL	484	-
**Sn**	0.23	<DL	2.38	0.23	0.07	0.75	ns
**Sr**	19	3	99	22	6	65	ns
**Te**	<DL	<DL	0.0148	<DL	<DL	0.0085	-
**Ti**	14	2	253	16	2	191	ns
**Tl**	0.0056	<0.0017	0.0262	0.0051	0.0016	0.0154	ns
**U**	0.030	0.002	0.204	0.028	0.005	0.120	ns
**V**	0.7	<DL	12.0	1.1	0.1	7.7	ns
**W**	<DL	<DL	0.0462	<DL	<DL	0.0156	-
**Zn**	86	<DL	451	89	39	300	ns
**Zr**	0.39	0.01	6.29	0.43	0.02	6.05	ns

[a] Mann-Whitney test: ns = not significant at $p > 0.05$ and significant at $p < 0.05$ (*). Elements with a detection frequency percentage >DL lower than 80% were excluded from the statistical calculation: Al, As, B, Bi, Nb, Si, Te, and W.

International Journal of Environmental Research and Public Health

MDPI

Table S6. Influence of fish consumption on element levels (mg kg^{-1}) in children's hair.

	No fish consumption (n=33)			Fish consumption (n=47)			
**Element**	**GM**	**Min**	**Max**	**GM**	**Min**	**Max**	***p*-value**
**Al**	2	<DL	233	1	<DL	167	-
**As**	0.03	<DL	0.65	0.04	<DL	0.32	-
**B**	1.1	<DL	5.1	1.3	<DL	29.2	-
**Ba**	7	<DL	73	14	<DL	74	**
**Be**	0.006	<DL	0.173	0.010	<DL	0.181	ns
**Bi**	0.001	<DL	0.551	0.001	<DL	0.076	-
**Ca**	1210	<DL	10700	1740	<DL	17300	ns
**Cd**	0.10	<DL	1.58	0.10	0.01	0.91	ns
**Ce**	0.8	0.1	13.4	1.2	0.03	12.9	ns
**Co**	0.25	0.02	3.23	0.39	0.05	3.58	ns
**Cr**	0.5	0.1	6.1	0.6	<DL	6.5	ns
**Cs**	0.011	<DL	0.156	0.015	<DL	0.150	ns
**Cu**	12.9	7.6	32.7	10.7	4.9	29.8	ns
**Fe**	192	17	3730	296	11	4000	ns
**Ga**	0.06	0.01	1.00	0.09	0.005	1.07	ns
**Hg**	0.034	0.013	0.238	0.078	0.012	0.483	***
**K**	833	<DL	4420	1240	<DL	6690	ns
**La**	0.5	0.0	7.3	0.6	0.01	6.6	ns
**Li**	0.17	0.02	1.34	0.22	0.02	1.60	ns
**Mg**	241	60	1470	316	44	2320	ns
**Mn**	22	1	178	33	2	184	ns
**Mo**	0.04	<DL	0.89	0.10	<DL	1.53	*
**Na**	1670	301	7450	1890	160	15000	ns
**Nb**	0.0005	<DL	0.80	0.0013	<DL	0.88	-
**Ni**	1.3	0.3	6.8	1.2	0.2	8.8	ns
**P**	76	<DL	455	114	21	611	*
**Pb**	4.0	0.7	32.8	2.8	0.3	26.6	ns
**Rb**	1.1	0.2	7.3	1.5	0.1	8.6	ns
**Sb**	0.08	<DL	8.85	0.07	<DL	0.62	ns
**Se**	0.21	<DL	0.67	0.18	<DL	0.58	ns
**Si**	39	<DL	499	94	<DL	484	-
**Sn**	0.22	<DL	1.05	0.24	0.04	2.38	ns
**Sr**	16	3	87	22	3	99	ns
**Te**	0.0013	<DL	0.0076	0.0014	<DL	0.0148	-
**Ti**	11	2	226	17	2	253	ns
**Tl**	0.0047	0.0018	0.0243	0.0060	0.0007	0.0262	*
**U**	0.024	0.002	0.167	0.033	0.005	0.204	ns
**V**	0.5	<DL	10.3	1.0	0.1	12.0	ns
**W**	0.0007	<DL	0.0462	0.0005	<DL	0.0210	-
**Zn**	105	<DL	451	76	<DL	300	**
**Zr**	0.29	0.01	4.58	0.49	0.02	6.29	ns

[a] Mann-Whitney test: ns = not significant at p >0.05, and significant at p <0.05 (*), p <0.01 (**), and p <0.001 (***). Elements with a detection frequency percentage >DL lower than 80% were excluded from the statistical calculation: Al, As, B, Bi, Nb, Si, Te, and W.

International Journal of
Environmental Research and Public Health

MDPI

Table S7. Results of the backward multiple linear regression model analysis for major and trace elements.

Elements	Factors	B[a]	SE[b]	[c]	*p*-value	95% Confidence interval for B Lower bound	Upper bound	R^2	Adjusted R^2 of the model[d]
Ca	Constant	8.133	0.276		<0.001	7.582	8.684	0.427	0.399
	Gender	-0.456	0.170	-0.259	0.009	-0.795	-0.116		
	Fruit and vegetables	-0.926	0.250	-0.362	<0.001	-1.426	-0.427		
	Drinking water	0.742	0.172	0.421	<0.001	0.398	1.086		
Ce	Constant	0.872	0.340		0.013	0.193	1.550	0.541	0.512
	Gender	-0.996	0.206	-0.420	<0.001	-1.408	-0.585		
	Fruit and vegetables	-0.857	0.304	-0.245	0.006	-1.464	-0.249		
	Drinking water	1.228	0.211	0.515	<0.001	0.807	1.649		
Co	Constant	-0.321	0.266		0.233	-0.852	0.211	0.618	0.600
	Gender	-0.833	0.162	-0.400	<0.001	-1.156	-0.510		
	Fruit and vegetables	-0.958	0.241	-0.312	<0.001	-1.439	-0.477		
	Drinking water	1.104	0.164	0.529	<0.001	0.776	1.433		
Cr	Constant	-0.496	0.180		0.008	-0.858	-0.135	0.255	0.212
	Age	-0.266	0.116	-0.284	0.026	-0.498	-0.033		
	Drinking water	0.628	0.170	0.457	0.001	0.287	0.968		
Cu	Constant	1.910	0.231		<0.001	1.448	2.373	0.158	0.131
	BMI	0.035	0.015	0.280	0.019	0.006	0.065		
	Passive smoking	-0.200	0.095	-0.244	0.040	-0.391	-0.009		
Fe	Constant	6.513	0.367		<0.001	5.780	7.247	0.593	0.567
	Gender	-0.947	0.210	-0.364	<0.001	-1.367	-0.526		
	Fruit and vegetables	-1.043	0.313	-0.272	0.001	-1.669	-0.417		
	Drinking water	1.632	0.247	0.625	<0.001	1.139	2.125		
Ga	Constant	-1.829	0.339		<0.001	-2.506	-1.152	0.548	0.526
	Gender	-0.883	0.206	-0.363	<0.001	-1.295	-0.472		
	Fruit and vegetables	-0.979	0.307	-0.273	0.002	-1.592	-0.366		
	Drinking water	1.270	0.209	0.520	<0.001	0.852	1.688		
K	Constant	7.521	0.194		<0.001	7.131	7.911	0.177	0.144
	Gender	-0.479	0.190	-0.328	0.015	-0.860	-0.097		
La	Constant	0.275	0.339		0.419	-0.401	0.952	0.541	0.512
	Gender	-1.008	0.205	-0.426	<0.001	-1.419	-0.598		
	Passive smoking	-0.566	0.273	-0.182	0.042	-1.112	-0.021		
	Fruit and vegetables	-0.869	0.303	-0.249	0.006	-1.474	-0.263		
	Drinking water	1.207	0.210	0.508	<0.001	0.787	1.627		
Mg	Constant	6.103	0.235		<0.001	5.634	6.573	0.468	0.443
	Gender	-0.615	0.143	-0.395	<0.001	-0.900	-0.330		
	Fruit and vegetables	-0.521	0.213	-0.227	0.017	-0.946	-0.095		
	Drinking water	0.705	0.145	0.451	<0.001	0.414	0.995		
Mn	Constant	4.325	0.287		<0.001	3.751	4.898	0.531	0.500
	Gender	-0.784	0.165	-0.415	<0.001	-1.114	-0.454		
	Fruit and vegetables	-0.819	0.245	-0.296	0.001	-1.308	-0.330		
	Drinking water	0.998	0.193	0.527	<0.001	0.613	1.383		
Mo	Constant	-1.981	0.253		<0.001	-2.489	-1.473	0.401	0.355
	Gender	-0.477	0.149	-0.347	0.002	-0.775	-0.178		
	Age	0.291	0.109	0.294	0.010	0.072	0.509		
	Fruit and vegetables	-0.520	0.219	-0.263	0.022	-0.960	-0.079		
	Drinking water	0.478	0.149	0.348	0.002	0.178	0.777		
Na	Constant	7.525	0.251		<0.001	7.025	8.026	0.172	0.146
	Gender	-0.682	0.235	-0.328	0.005	-1.151	-0.213		
	Age	0.349	0.164	0.241	0.037	0.022	0.675		
P	Constant	4.586	0.085		<0.001	4.416	4.755	0.278	0.254
	Gender	-0.209	0.099	-0.234	0.039	-0.407	-0.011		
	Drinking water	0.411	0.099	0.458	<0.001	0.212	0.610		
Rb	Constant	0.906	0.329		0.008	0.249	1.562	0.370	0.330
	Gender	-0.798	0.199	-0.407	<0.001	-1.196	-0.400		
	Drinking water	0.751	0.204	0.382	<0.001	0.344	1.159		
Se	Constant	-1.671	0.328		<0.001	-2.332	-1.010	0.209	0.154
	Age	-0.292	0.098	-0.459	0.005	-0.489	-0.095		
	Passive smoking	-0.455	0.185	-0.359	0.018	-0.827	-0.082		

**Sr**	**Constant**	3.562	0.219		<0.001	3.125	4.000	0.436	0.409
	**Gender**	-0.392	0.135	-0.278	0.005	-0.662	-0.123		
	**Fruit and vegetables**	-0.735	0.198	-0.358	<0.001	-1.132	-0.338		
	**Drinking water**	0.598	0.137	0.424	<0.001	0.325	0.870		
**Ti**	**Constant**	3.266	0.317		<0.001	2.632	3.900	0.567	0.547
	**Gender**	-0.722	0.193	-0.310	<0.001	-1.107	-0.337		
	**Fruit and vegetables**	-0.943	0.287	-0.274	0.002	-1.517	-0.369		
	**Drinking water**	1.327	0.196	0.567	<0.001	0.935	1.719		
**V**	**Constant**	0.643	0.273		0.022	0.097	1.189	0.641	0.624
	**Gender**	-0.855	0.167	-0.388	<0.001	-1.189	-0.521		
	**Fruit and vegetables**	-1.015	0.248	-0.314	<0.001	-1.510	-0.521		
	**Drinking water**	1.237	0.170	0.560	<0.001	0.899	1.576		
**Zn**	**Constant**	4.503	0.175		<0.001	4.154	4.852	0.398	0.369
	**Gender**	0.284	0.108	0.264	0.011	0.069	0.499		
	**Drinking water**	-0.529	0.109	-0.491	<0.001	-0.748	-0.311		
**Zr**	**Constant**	-0.050	0.385		0.898	-0.818	0.719	0.519	0.497
	**Gender**	-0.900	0.234	-0.335	<0.001	-1.367	-0.433		
	**Fruit and vegetables**	-1.152	0.348	-0.291	0.002	-1.848	-0.456		
	**Drinking water**	1.352	0.238	0.502	0.005	-0.872	-0.165		

No variable entered in the models run for Al, As, B, Bi, Nb, Pb, Sb, Si, Te, and W. [a] B, non-standardized regression coefficients. [b] SE, standard error. [c] β, standardized regression coefficients. [d] Constant, estimated intercept value. Variables considered: gender (female = 0, male = 1), age categorized as <5 years old (=0), 6-11 years old (=1) or 12-18 years old (=2), body mass index (BMI) categorized as underweight (=0), normal weight (=1) or overweight/obesity (=2), drinking water (bottle and/or improved water = 0, Blue Nile river water = 1), fish consumption (no = 0, yes = 1), fruit and vegetables consumption (no = 0, yes = 1), and cereals consumption (g month^{-1}, continuous variable).

International Journal of
Environmental Research and Public Health

MDPI

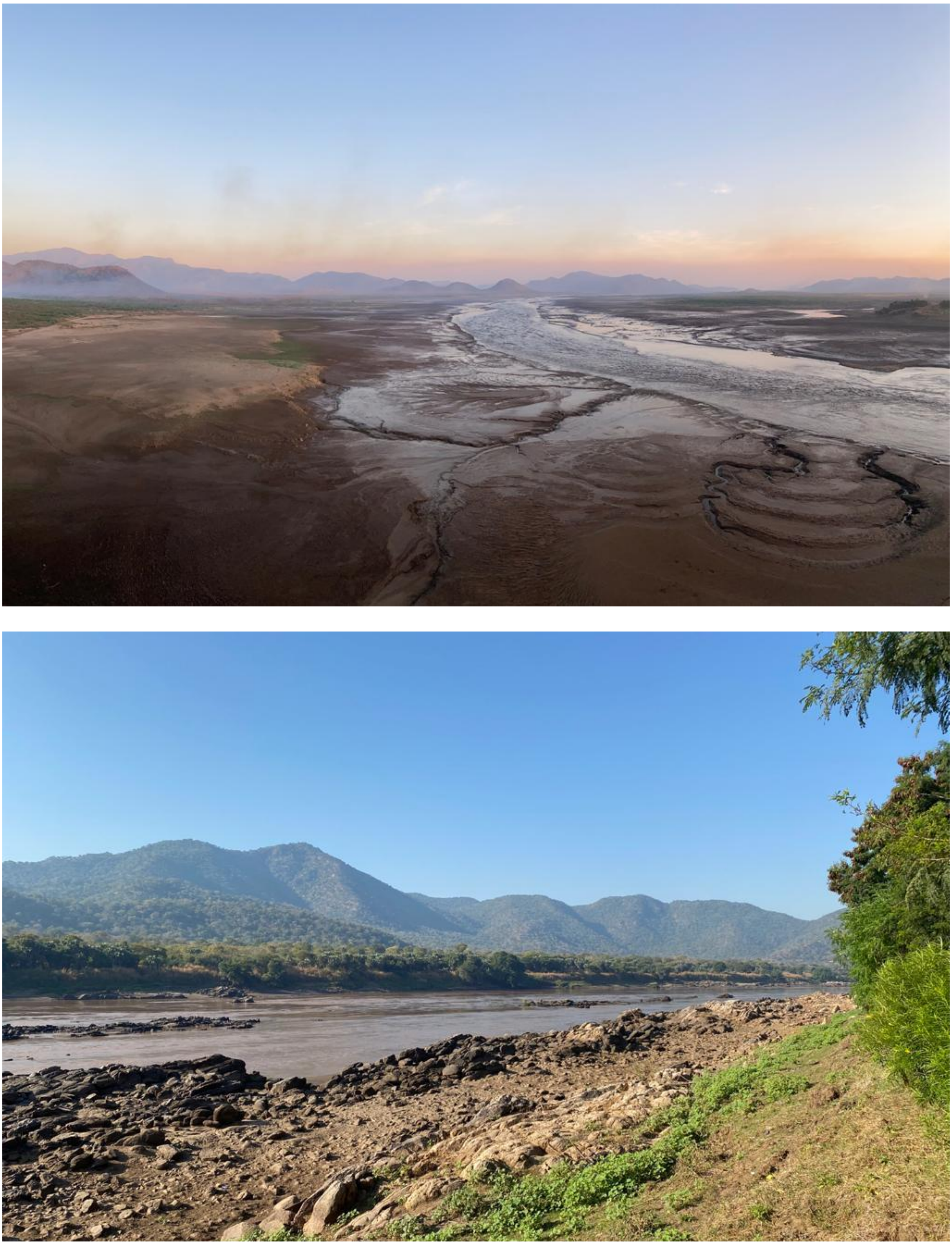

Figure S1. Landscapes of the studied area (Bameza in the Benishangul-Gumuz region along the Blue Nile in north-western Ethiopia, Africa).

International Journal of
Environmental Research and Public Health

MDPI

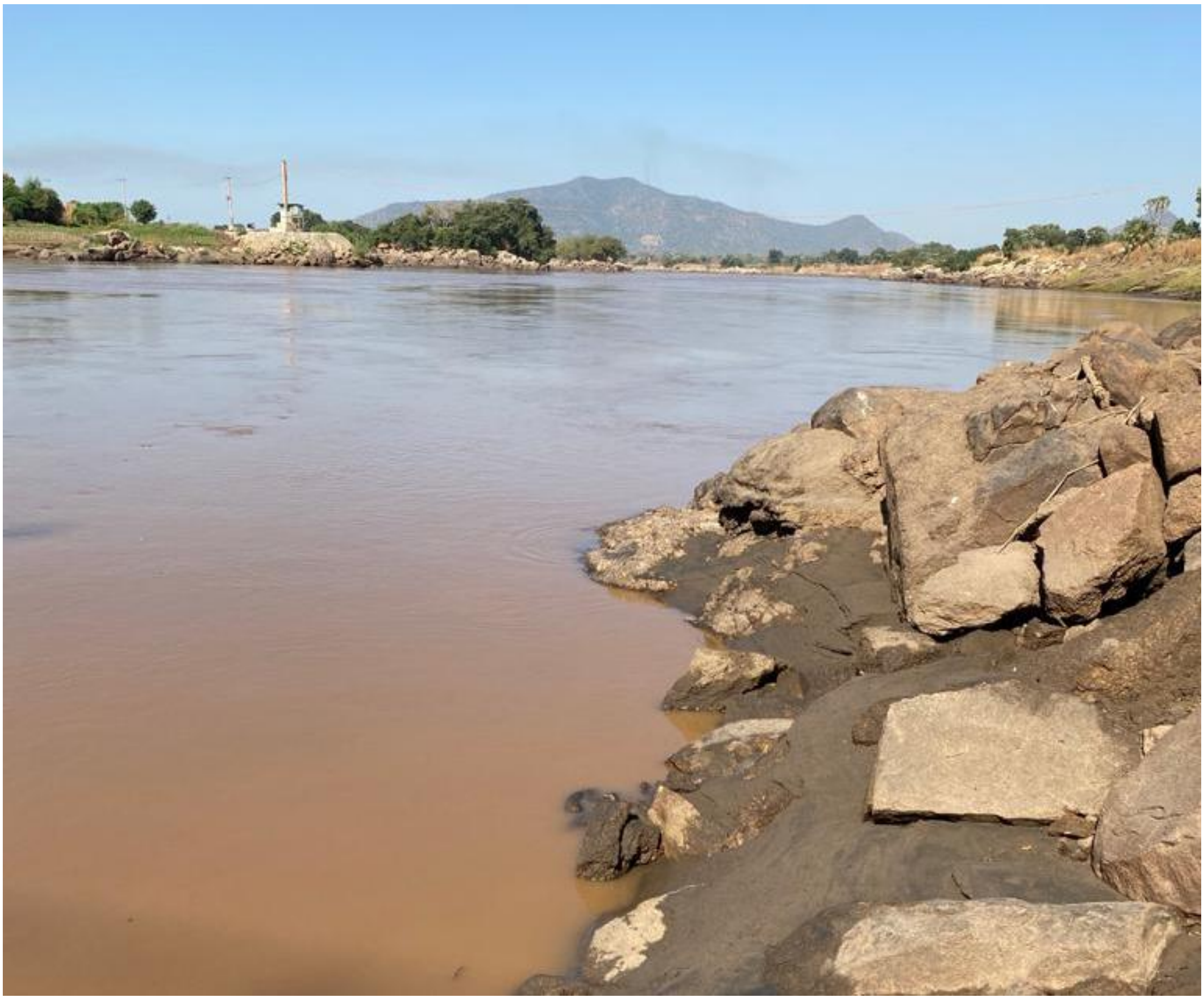

Figure S2. Sampling site of the Blue Nile river water (Bameza, Ethiopia).

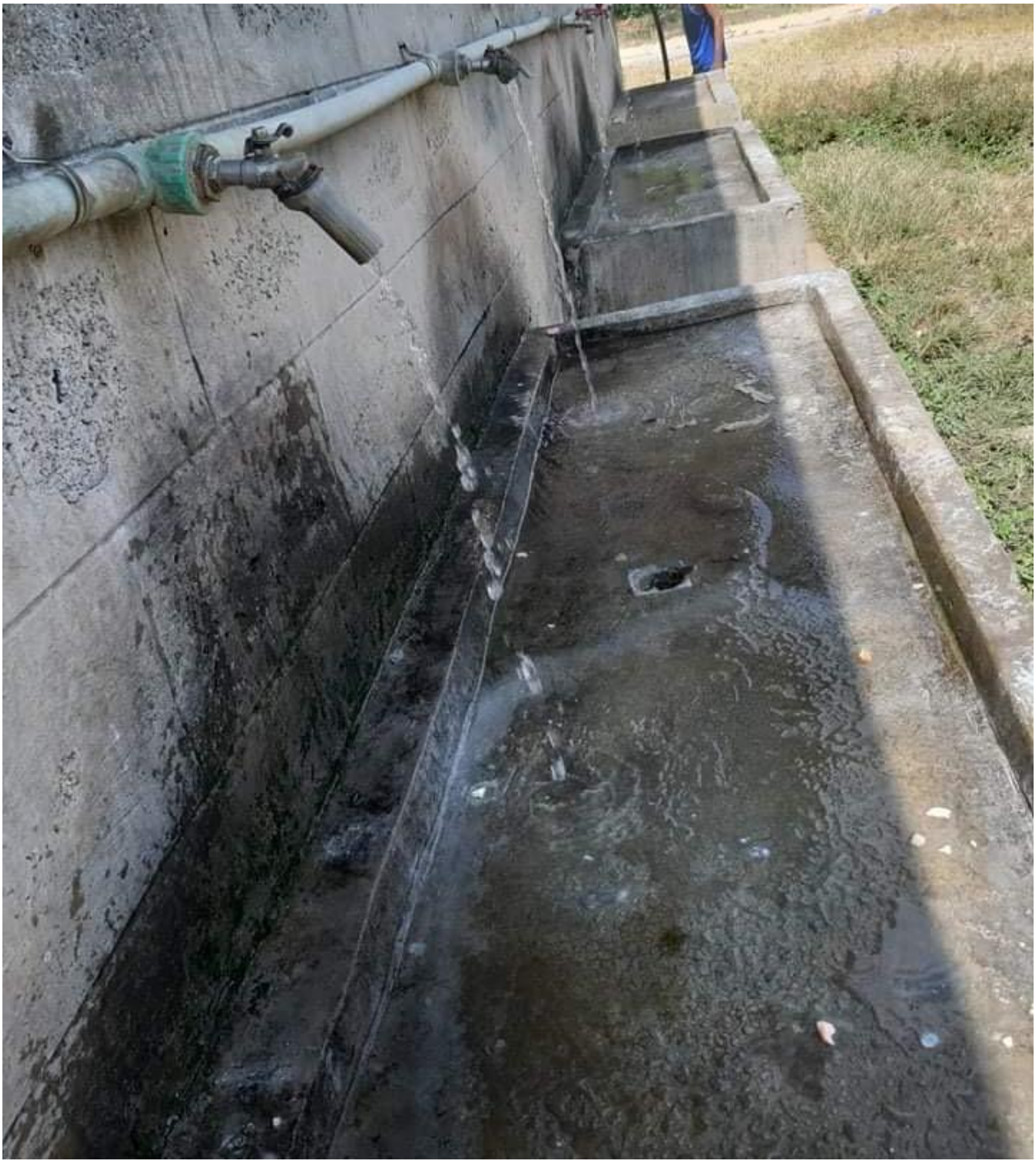

Figure S3. Sampling site of the treated water of the Blue Nile river (Bameza, Ethiopia).
